# Maladaptive migration behaviour in hybrids links to predator‐mediated ecological selection

**DOI:** 10.1111/1365-2656.13308

**Published:** 2020-08-23

**Authors:** Varpu Pärssinen, Kaj Hulthén, Christer Brönmark, Christian Skov, Jakob Brodersen, Henrik Baktoft, Ben B. Chapman, Lars‐Anders Hansson, Per Anders Nilsson

**Affiliations:** ^1^ Department of Biology ‐ Aquatic Ecology Lund University Lund Sweden; ^2^ National Institute of Aquatic Resources Technical University of Denmark (DTU) Silkeborg Denmark; ^3^ Department of Fish Ecology and Evolution Center for Ecology, Evolution and Biogeochemistry EAWAG Swiss Federal Institute of Aquatic Science and Technology Kastanienbaum Switzerland; ^4^ Department of Aquatic Ecology & Evolution Institute of Ecology and Evolution University of Bern Bern Switzerland; ^5^ Division of Evolution and Genomics School of Biological Sciences University of Manchester Manchester UK; ^6^ Department of Environmental and Life Sciences ‐ Biology Karlstad University Karlstad Sweden

**Keywords:** fish, hybrid viability, partial migration, predator–prey, species integrity

## Abstract

1. Different migratory species have evolved distinct migratory characteristics that improve fitness in their particular ecological niches. However, when such species hybridize, migratory traits from parental species can combine maladaptively and cause hybrids to fall between parental fitness peaks, with potential consequences for hybrid viability and species integrity.
2. Here, we take advantage of a natural cross‐breeding incident to study migratory behaviour in naturally occurring hybrids as well as in their parental species and explore links between migratory traits and predation risk.
3. To achieve this, we used electronic tags and passive telemetry to record detailed individual migration patterns (timing and number of migratory trips) in two common freshwater fish species, roach *Rutilus rutilus*, common bream *Abramis brama* as well as their hybrids. Next, we scanned for tags regurgitated by a key avian predator (great cormorant *Phalacrocorax carbo*) at nearby roosting sites, allowing us to directly link migratory behaviour to predation risk in the wild.
4. We found that hybrid individuals showed a higher number of short, multi‐trip movements between lake and stream habitats as compared to both parental species. The mean date of first lake departure differed between bream and roach by more than 10 days, while hybrids departed in two distinct peaks that overlapped with the parental species' averages. Moreover, the probability of cormorant predation increased with multi‐trip movement frequency across species and was higher for hybrids.
5. Our data provide novel insights into hybrid viability, with links to predator‐mediated ecological selection. Increased exposure to predators via maladaptive migratory behaviour reduces hybrid survival and can thereby reinforce species integrity.

## Graphical abstract

The study provides direct empirical support for predator‐mediated ecological selection against hybridization by identifying a mechanism for the higher avian predation risk of hybrids between two cyprinid species, as well as illustrating that hybrids can express novel and intermediate migratory behaviours in relation to their parental species.

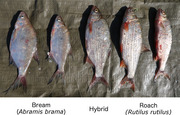

## 1 INTRODUCTION

Each year, millions of animals migrate to pursue spatiotemporal variation in suitable environmental conditions and seek shelter from natural enemies, such as parasites and predators (Chapman et al., 2014; Dingle, 2014). Key migratory traits, including timing, duration and distance, vary not only across the animal kingdom but also between closely related species (Pulido, 2007). Thus, natural selection seems to have moulded highly species‐specific migratory syndromes with a suite of specifically adapted morphological, behavioural and physiological adaptations. While migratory behaviour can vary both across (Altizer & Davis, 2010; Chamberlain, Bensch, Feng, Åkesson, & Andersson, 2000) and within (Secor, 1999; Skov et al., 2011) populations, each species should have a pool of available migratory behaviours that have evolved according to their specific ecological niche, as there is evidence that genetic changes can be linked to even relatively small changes in migratory behaviour (Kovach, Gharrett, & Tallmon, 2012). For example, different salmonid species have evolved their own suites of intraspecific migratory phenotypes, and their expression depends on traits such as body size and growth rate of the individual (Dodson, Aubin‐Horth, Thériault, & Páez, 2013). Key migratory traits, such as timing, can have far‐reaching implications for individual fitness as successful migration hinges on the ability to exploit or escape temporary recourses and threats as they arise. Migrants arriving too early may face unfavourable environmental conditions at their destination, whereas late migrants may have to cope with intense competition for resources and/or mating opportunities (Bêty, Giroux, & Gauthier, 2004; Marra, Hobson, & Holmes, 1998). Migratory journeys can also be energetically demanding and incur substantial cost of transport (Hansson & Åkesson, 2014; Johansson, Muijres, & Hedenström, 2014), which may explain differences in migration propensity and distance migrated. Moreover, in short‐distance migration, individuals may have the opportunity to undertake numerous trips between winter and summer habitats. Such multi‐trip migration has been described as a behavioural continuum between the stereotyped full residency and definite migration in ungulates (Cagnacci et al., 2011), despite not receiving much focus in other systems, and may be common in nature. Increased multi‐trip migration frequency could, however, come with a cost (e.g. use of energy or exposure to predators), and hereby result in reduced fitness.

In nature, closely related species, including migratory species, may hybridize and produce fertile offspring. Unless there is sufficient endo‐ or exogenous selection against hybridization, such species may collapse into hybrid swarms (Seehausen, Van Alphen, & Witte, 1997; Taylor et al., 2006; Zhang, Thibert‐Plante, Ripa, Svanbäck, & Brännström, 2019). Commonly, ecological selection against hybrids arises if hybrid offspring have phenotypes that fall between the adaptive peaks of their parental species (Hatfield & Schluter, 1999; Martin & Wainwright, 2013; Nosil, 2004). Thus, the relative hybrid inviability would contribute to species integrity by reducing the likelihood of introgression between parental species (Nosil & Crespi, 2006). Natural cross‐breeding incidents involving parental species and their fertile hybrids present an excellent scenario to test if hybrids express divergent traits that are ecologically selected against.

Contemporary studies of migratory behaviour in hybrids have reported either migratory routes that closely match, or, alternatively, are intermediate relative to parental species (Delmore & Irwin, 2014; Helbig, 1991; Moore et al., 2010; Väli, Mirski, Sellis, Dagys, & Maciorowski, 2018). As species can be subjected to predation during migration (Hebblewhite & Merrill, 2007; Schmaljohann & Dierschke, 2005), divergent migratory behaviour expressed by hybrids would be expected to lead to ecological incompatibility, manifested via higher predation mortality. However, whether hybrid migratory traits can be directly selected against by predators remains to be tested.

Freshwater fishes have emerged as an important model system to study the causes and consequences of animal migration (Brönmark et al., 2014; Chapman et al., 2012; Lucas & Baras, 2001). The roach *Rutilus rutilus* and the common bream *Abramis brama* are common freshwater fishes that migrate from shallow lakes into connected streams during the winter season (Skov, Brodersen, Nilsson, Hansson, & Brönmark, 2008). Their migratory dynamics are shaped by temperature‐driven changes in the trade‐off between predation risk and growth potential (Brönmark, Skov, Brodersen, Nilsson, & Hansson, 2008). The stream wintering grounds hold low densities of piscivorous and avian predators, and the large majority (over 90%) of individuals get predated when situated in the lake habitat (Skov et al., 2013), which means that predation risk varies between the lake (high predation) and the streams (low predation). During winter, migrants thus benefit from a reduced predation risk by refuging in the low‐risk streams (Skov et al., 2013), but have to pay a foraging cost by migrating to this comparably food‐deprived habitat (Brodersen, Nilsson, Hansson, Skov, & Brönmark, 2008; Chapman et al., 2013). The importance of predation risk for migratory decision‐making in cyprinid fish is further highlighted by the fact that individual migratory propensity and duration is linked to predation vulnerability, where migrating individuals experience significantly lower risk of cormorant predation (Skov et al., 2011), and can be facultatively induced via experimental manipulations to perceived predation risk (Hulthén et al., 2015). Furthermore, it was recently demonstrated that natural hybrids between roach and bream suffer around 2–4 times as high risk of predation from great cormorants *Phalacrocorax carbo* compared to parental species (Nilsson et al., 2017). However, the link between migratory behaviour and higher vulnerability to predation in hybrids remains to be evaluated.

In this study, we used passive telemetry to survey and link the expression of migratory traits of roach, bream and their hybrids to the risk of cormorant predation at the level of individuals. We predicted that hybrids would express a divergent migratory behaviour relative to their parent species and that this would incur a fitness cost through increased susceptibility to predation.

## 2 MATERIALS AND METHODS

### 2.1 Study species

Members of the Cyprinidae have the highest frequency of hybridization among all groups of freshwater fishes (Scribner, Page, & Bartron, 2000) and thus constitute an exceptional empirical substrate to test for hybrid inviability as a result of divergent migratory traits. Roach and common bream are two closely related freshwater cyprinid fish species that readily form fertile (Wood & Jordan, 1987) and morphologically (Wood & Jordan, 1987) and ecologically (Toscano et al., 2010) intermediate hybrids. The two species have external fertilization, and spawning periods overlap temporally and spatially (Kottelat & Freyhof, 2007), facilitating natural hybridization.

### 2.2 Study system, electronic tagging and migration monitoring

Individual migration patterns of roach, bream and their hybrids were quantified by passive telemetry. At the site of our study, year 2005 had an exceptional peak in roach × bream hybrid prevalence (9.8%) in the yearly electrofishing surveys in comparison to later years (mean 1.31%, range = 0%–6.53%). During 2005, between 30 September and 17 October, we electrofished and seined roach, bream and their hybrids in the Danish lake Loldrup Sø (56°29′N, 9°26′E), a small, shallow and slightly eutrophic lake (area 39 ha; average depth 1.2 m; mean summer Secchi depth 1.1 m) that has one inlet and one outlet stream. The fish community of Loldrup Sø is dominated by roach and common bream but also includes the piscivorous species pike *Esox lucius*, Eurasian perch *Perca fluviatilis* and pikeperch *Sander lucioperca*. Following capture, we measured the total length (nearest mm) and weight (nearest 0.1 g) of all individuals, and identified the two species and their hybrids from distinct morphological characteristics (e.g. body shape, fin, eye and general colours, see Nilsson et al., 2017 for photos and details). Fish body size may influence the probability of cormorant predation, as well as migration propensity through predator prey‐size selectivity (Skov et al., 2013) and gape‐size limits (Skov et al., 2011), so to minimize this variation between groups we evaluated roach and bream individuals that were similar to the size range of caught hybrids [roach: 198–286 mm (234.4 ± 24.3 mm, *M* ± *SD*), *n* = 53, bream: 201–320 mm (243.8 ± 30.1 mm), *n* = 31, hybrids: 202–285 mm (234.2 ± 17.9 mm), *n* = 58]. Fish were then individually tagged by surgically implanting a uniquely coded TIRIS Passive Integrated Transponder (PIT) tag (Texas Instruments, RI‐TRP‐RRHP, half duplex, 134 kHz, 23.1 mm long, 3.85 mm diameter, 0.6 g in air) into the body cavity. This method of PIT tagging has no observable effects on survival or body condition in cyprinids (Hulthén et al., 2014; Skov et al., 2005, 2020). Migratory patterns of individual migrants were monitored using stationary, continuously operating antenna arrays. Two loop‐shaped antennas, each covering the entire cross‐section of the streams, were placed near the inlet/outlet 3–6 m apart in both streams connected to the lake. When a tagged fish swims through or in the vicinity of an antenna, the tag is energized and emits a unique identity code. The RFID multiplexer units record migratory behaviour at the individual level by storing all tag detections (individual IDs) together with a date and time stamp on a memory card. The use of paired antennas enables determination of fish swimming direction based on the sequence of detections. The recording frequency was set to 5 energize/receive cycles per second.

**Figure 1:**
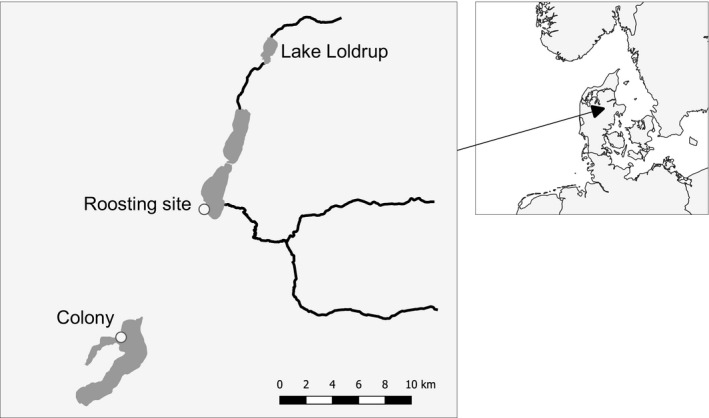
Map of the study site (Danish lake Loldrup Sø) and nearby cormorant sites that were scanned for PIT tags

To identify predated individuals after natural predation had time to occur, we performed extensive scans for PIT tags (Nilsson et al., 2017; Skov et al., 2013) in 2008 and 2009 (nearly weekly scans between 25 March–22 July 2008, and 3 December 2008–2 November 2009, on 70 separate days) at a nearby breeding colony and a roosting site for cormorants (Figure 1), which are major local predators of cyprinids. Additionally, we scanned a cormorant nesting colony located 5–12 km from the lake (depending on position in the study lake). A distant nesting colony (39 km away) and roosting place (27 km away) were also scanned on several occasions, but no tags were recovered from these locations. When scanning for tags (systematically sweeping the whole area under roosts and colonies along predefined transects), operators used battery‐powered (12 V, 7.2 Ah) and portable flat‐plate scanner systems with circular antennas (see Nilsson et al., 2017 for details). PIT tags remain functional and at the site of deposition for several years, as evidenced by a high retrieval rate (97%) of these tags also during scannings performed in 2015 (i.e. 7–8 years after initial scannings). Furthermore, no additional tags originating from this study were detected during these scannings.

### 2.3 Data handling and analyses

We quantified two key migratory traits for roach, bream and their hybrids: the number of migratory trips between the lake and streams during the season, and the timing of individuals' first lake departure. We predicted that habitat shifts during the migration period would link to susceptibility to predation, as movement between lake and stream habitats would expose individuals to cormorant predation. To exclude erratic, small‐scale movements in the vicinity of an antenna, only cases where the fish passed both antennae and stayed in the new habitat (i.e. lake or stream) for over 30 min were included in the analyses. The number of migratory trips between the stream and lake was recorded from 6 October to 31 December in 2005. The end of December was chosen as a limit as both species and hybrids showed high levels of migration activity throughout the autumn season but dramatically reduced activity after the end of the year for the following winter months. In these systems, individual migratory decisions are highly consistent, with the majority (nearly 90%) of individuals not shifting their migratory strategy between years (i.e. switch from migration to residency and back again), which suggests that cases where an individual migrated in one year but not the next is most likely due to mortality rather than migratory plasticity (Brodersen et al., 2014). Hence, we considered fish that migrated at least once but did not show any activity in the following spring or autumn to have died during winter. When comparing the number of migration trips among species (including hybrids), we thus only included data from individuals registered as migrants the year after data were collected, to enable evaluation of individuals that with certainty were alive during the entire migration period evaluated. For analysis concerning timing of first lake departure, we used all individuals that migrated at least once. To evaluate the effect of the number of migration trips during the migration period on the probability of cormorant predation, we needed a standardized measure of migration frequency. Thus, to account for the obviously shorter observation period in predated individuals as well as to compensate for individual differences in first departure date, we calculated the length of the observation period for each individual. Each individual observation period was defined as the number of days between the first and last detection by antennae for perished individuals, and between first detection and the last day of the evaluated period (31 December) for individuals confirmed to be alive the following year. Individual migration frequency was then calculated for each individual by dividing the individual number of recorded migration trips with the number of days of the individual migration period. Individuals that did not migrate after tagging received a value of zero for their observation period and migration frequency. Overall migration activity in the population was relatively constant throughout the observation period, so shorter observation period was not linked to higher migration frequency.

The differences between roach, bream and hybrid individuals in the number of migration trips during the whole migration period were evaluated with a GLM (Poisson distribution with a log‐link function) followed by a post‐hoc Tukey's test. Initiation of migration, that is, ordinal dates of first recorded migration activity for individuals, was analysed with a Kruskal–Wallis test, with post‐hoc pairwise comparisons between species and hybrids with Wilcoxon rank sum tests with Benjamini and Hochberg correction. The effect of migration frequency on the individual probability of being predated by a cormorant was estimated with logistic regression (GLM, binomial distribution with a logit‐link function) predicting the probability of being predated by a cormorant. The full model evaluating individuals' fates (predated or not) included species (roach, bream or hybrid), individual body length, first day of migration (as number of days since 1 September), migration frequency, the interaction term between species and migration frequency, and observation period length. To include all individuals in this analysis, the individuals that never migrated received a period‐length ceiling value of 122 (number of days from 1 September to 31 December). Observation period length was always included when comparing models to compensate for the possible effects of observation period length on migration frequency and predation risk. The full model was reduced by ranking possible models by AIC values with dredge command (mumin package in r; Barton, 2019). All analyses were run in r software (version 3.5.1).

## 3 RESULTS

**Figure 2:**
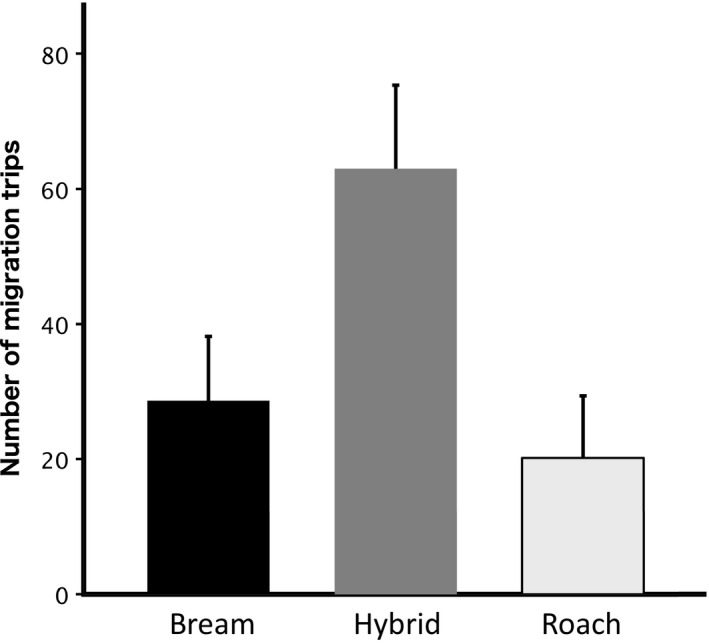
Estimated mean (±95% CI) number of migration trips over the migration season for bream, roach and their hybrids

**Figure 3:**
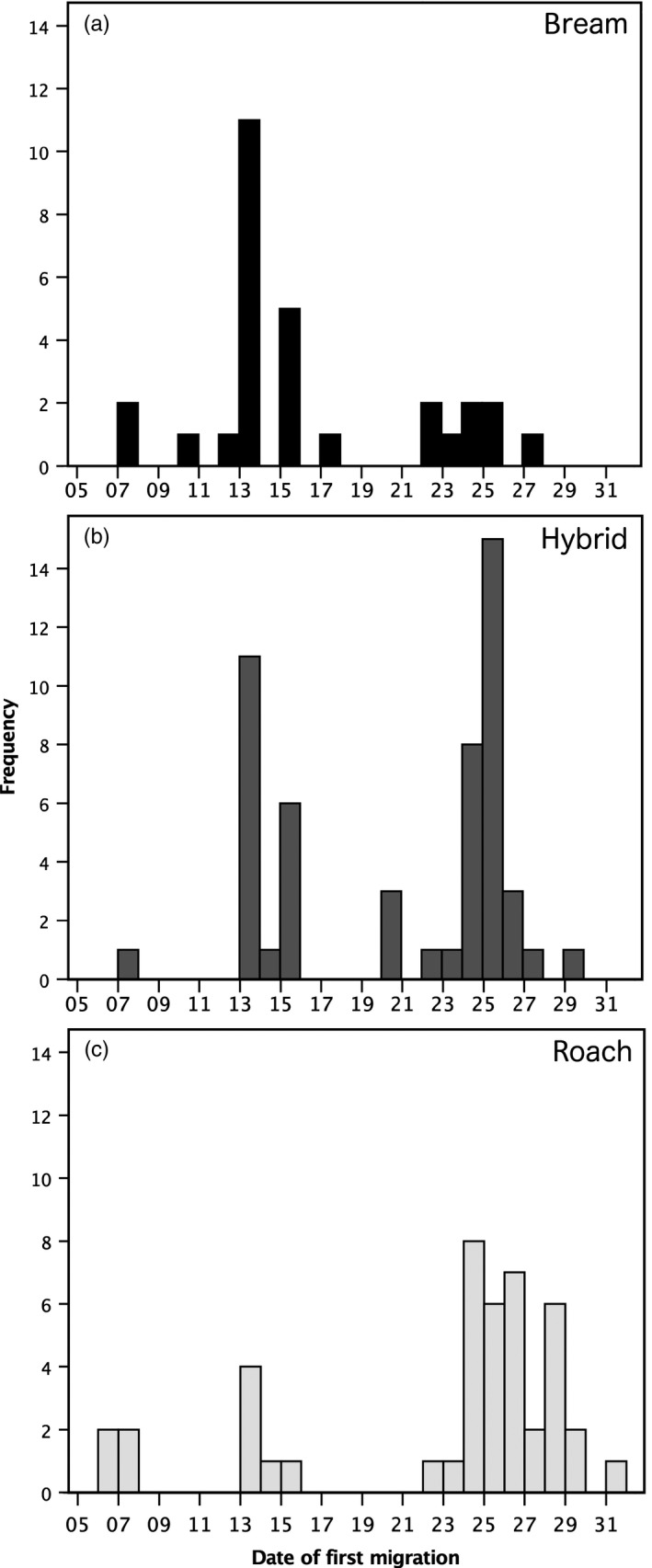
Frequency distribution showing timing of initiation of migration (first trip from lake to the stream habitats) in (a) bream, (b) hybrids and (c) roach. Dates refer to October 2005

**Figure 4:**
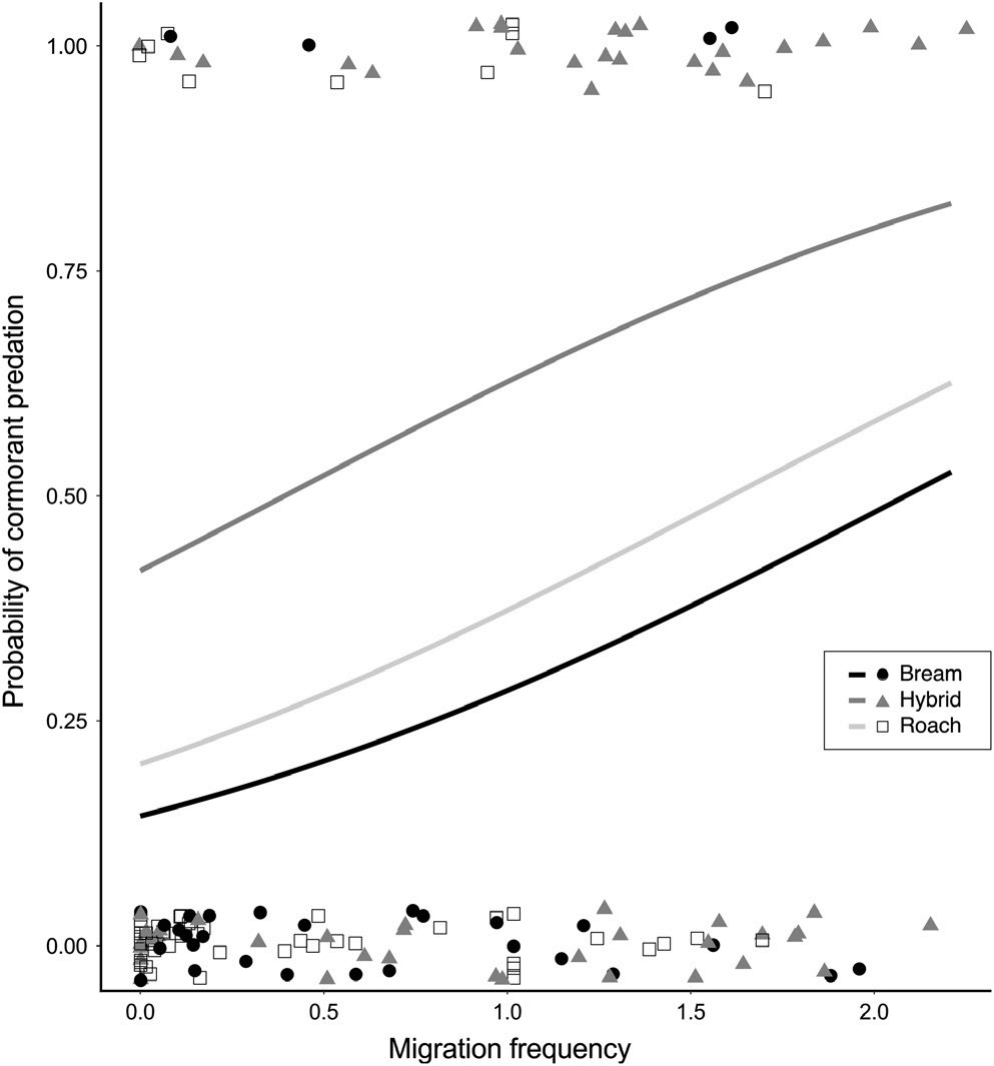
Effect of migration frequency on the probability of cormorant predation for bream, roach and their hybrids. Curves visualize probability distributions predicted by a GLM (binomial, logit‐link) model on individual fish migration frequencies and fate [predated (1) or not (0)], denoted by jittered raw data points

All 142 tagged and released individuals were used in the analyses estimating the probability of cormorant predation. A total of 133 individuals migrated and were thus used for analysis of first migration date. Out of these, 80 individuals also showed activity during the following year and were thus used to compare the number of migration trips among species and hybrids. The number of migration trips between the habitats over the season differed among species and hybrids [χ^2^ = 813.93, *p* < 0.001, *n* = 16 (bream), 37 (hybrid), 27 (roach)], with hybrids showing a higher number of migration trips as compared to both roach and bream (*p* < 0.001 in both comparisons) but the parental species did not differ significantly from each other (*p* = 0.069, Figure 2). The roach, bream and their hybrids initiated their peak migration activity at different times [χ^2^ = 20.559, *p* < 0.001; Wilcoxon: bream‐roach *p* < 0.001, bream‐hybrid *p* = 0.0016, roach‐hybrid *p* = 0.0176; *n* = 29 (bream), 52 (hybrid), 44 (roach)]. Bream showed a stronger early peak in first migration (13–15 October) while roach showed a stronger late peak (24–26 October), while hybrids started their migration in two distinct peaks that overlapped with both bream and roach (Figure 3). The final GLM model with best prediction of probability of cormorant predation [AIC = 154.4 (AIC ranged 153.4–154.4 for preceding models), *n* = 31 (bream), 58 (hybrid), 53 (roach)] included migration frequency (χ^2^ = 7.573, *p* < 0.01) and species (χ^2^ = 6.6778, *p* = 0.023), with observation period included as a nonsignificant compensated effect (χ^2^ = 0.9944, *p* = 0.319). Hybrids and individuals (from all three fish categories) expressing a high frequency of migration trips were more likely to be predated by cormorants (Figure 4).

## 4 DISCUSSION

We here show that hybrids can differ from their parental species in migratory behaviours, and that migratory traits link to predation costs in the wild. The roach × bream hybrids are generally fertile (Wood & Jordan, 1987), and, hence, if there are no associated costs of hybridization, parental species may collapse into hybrid swarms upon hybrid back‐crossing (Taylor et al., 2006). Hence, hybrid inviability, here manifested as migration‐ and predator‐mediated survival costs, should be central for species integrity in our study system, and most likely so also in others. The novel link between maladaptive, multi‐trip migration behaviour in roach × bream hybrids and their established (Nilsson et al., 2017) higher risk of cormorant predation thus contributes to furthered understanding of processes promoting, for example, species integrity and ecological speciation (Gow, Peichel, & Taylor, 2007; Nosil, 2012).

The hybrids in our study showed a mixture of intermediate and novel behavioural migration patterns compared to their parental species. The mean initiation of migration (lake departure dates) differed between parental species with bream showing a stronger early peak in migratory activity as compared to roach, that showed a stronger late peak. Hybrids expressed a mixture of these migratory strategies, with one early and one late peak in onset of outmigration, each coinciding with the peaks of the parental species. If this is due to a genetic underpinning with contributions from both parental strategies in hybrids, or if hybrids simply behaviourally follow the peaks of the parental species, lies beyond interpretation from the present data, but our results are in line with the few previous studies tracking the migration behaviour of hybrids and their parental species (Delmore & Irwin, 2014; Moore et al., 2010).

We evaluated the number of multi‐trip migrations, which was found to be relatively low and comparably expressed in both of the parental species, whereas their hybrids showed a high propensity for multiple habitat shifts between lake and stream habitats. Consequently, hybrids express a distinctly different behavioural phenotype as compared to their parental species regarding multi‐trip migration behaviour. Such behaviour can convey several fitness costs including, for example, higher energy expenditure associated with migration (Brodersen, Nilsson, Ammitzbøll, et al., 2008) or suboptimal habitat choice (reduced foraging opportunity; Chapman et al., 2013). However, the ultimate fitness cost of mortality, here evaluated as probability of cormorant predation, should be regarded as a major cost of maladaptive, multi‐trip migration behaviour in our study system. Migratory movements can attract predators around critical migration corridors (Greenstreet, Morgan, Barnett, & Redhead, 1993; Jepsen, Pedersen, & Thorstad, 2000), so individuals with higher migration frequency may be more likely to be detected by predators, as is probably the case for hybrids in our study system. Furthermore, a significant fraction (up to 70%–80%) of the cyprinid populations can reside in the streams during winter (Chapman et al., 2015; Skov et al., 2011). By deviating from the norm, migrants that leave the streams multiple times during the winter season would thus form a small fraction of the potential prey in the lake habitat. Hence, the per capita risk for individuals that visit the lake habitat during winter would be high and such individuals would not be able to benefit from a predator dilution effect (Harts, Kristensen, & Kokko, 2016). We view the above as candidate mechanisms behind the elevated risk of predation for individuals with high migration frequency, and thereby particularly so for hybrids.

The maintenance of species' separate genetic identities can be enforced by a variety of mechanisms, including pre‐zygotic habitat and temporal isolation, immigrant inviability, behavioural isolation, as well as post‐mating pre‐zygotic barriers (Coyne & Orr, 2004; Rogers & Bernatchez, 2006; Rundle & Nosil, 2005). In the absence of pre‐zygotic hybridization barriers, post‐zygotic hybrid infertility or inviability could reduce gene flow between species (Coyne & Orr, 2004; Nosil, 2012). The migration‐mediated increased susceptibility to predation in hybrids, shown in the present work, contributes to the understanding of hybrid inviability, and thereby to the understanding of maintenance of species integrity. In recent years, there has also been an increased interest in hybridization as a process promoting increased genetic variation resulting in adaptive diversification (Marques, Meier, & Seehausen, 2019; Seehausen, 2004) and adaptation to human‐disturbed environments (Oziolor et al., 2019). The new variation can lead to novel phenotypes with new fitness peaks, and this increased hybrid fitness may, in turn, lead to either a new separate species or the collapse of the parental species (Abbott et al., 2013; Nolte & Tautz, 2010). The hybrids in our study exhibited a novel phenotype, which comes with fitness costs in the context of our study system, where roach, bream and great cormorants have coexisted for hundreds of years. As these species co‐occur throughout major parts of continental Europe, similar processes may be key to species integrity also in other systems. However, in areas where roach and bream are recently introduced, the hybrid phenotype seems to be more successful (Toscano et al., 2010), possibly due to lower predation pressure from cormorants in these systems. This indicates that the usual low occurrence of wild roach × bream F2 hybrids (Wyatt, Pitts, & Butlin, 2006) cannot be explained by lower hybrid fertility (Wood & Jordan, 1987), but, rather, implies additional ecological selection against hybrids. Although the occurrence of hybrids was relatively high on the year of our study, their abundance has remained relatively low in the following years, further supporting selection against hybrids in our system. As our study shows that predation risk is higher for hybrids across migration frequencies, ecological selection may occur also via other mechanisms. For example, intermediate body morphology and potentially disparate general behaviour of hybrids may reduce their escape performance (Domenici & Blake, 1997) upon predator attack as well as ability for energy intake during foraging to acquire enough resources to be able to reside in the low‐food stream habitats for longer time periods (Henning, Machado‐Schiaffino, Baumgarten, & Meyer, 2017), posing additional possible selection against hybrids.

Hybrids in our system show disparate (Figure 2) and intermediate (Figure 3) behaviours regarding migration onset and the number of multi‐trip migrations, respectively, compared to parental species. The mechanistic reasons behind these hybrid traits lie beyond the current data, but studies into, for example, genetic precursors and hybrid energy accumulation capacities may further our understanding of the underlying causes of these results. We conclude that although hybrids between roach and bream are fertile and thereby may potentially interfere with species integrity of the parental species, an increased exposure to predators via maladaptive migratory behaviour reduces hybrid survival and thereby acts as to conserve the parental species. Such mechanistic understanding of hybrid maladaptive migration behaviour, with links to predator‐mediated ecological selection against hybrids, holds promising keys to furthered understanding of species integrity and speciation processes in the wild.

## AUTHORS' CONTRIBUTIONS

C.S. and H.B. collected field data; V.P. analysed the data and prepared the figures; V.P., K.H., C.B., C.S., J.B., H.B., B.B.C., L.‐A.H. and P.A.N. contributed substantially to the design, drafting and revisions of the study. All authors approved the final version of the manuscript and agree to be held accountable for the contents.

## Data Availability

The data that support our findings in this study and r code used to analyse those data are available online at Dryad Data Repository at http://doi.org/10.5061/dryad.73n5tb2tw (Pärssinen et al., 2020).
